# Neoadjuvant carboplatin in triple-negative breast cancer: results from NACATRINE, a randomized phase II clinical trial

**DOI:** 10.1007/s10549-023-07011-0

**Published:** 2023-08-14

**Authors:** Cristiano de Pádua Souza, Ana Suellen Barroso Carneiro, Ana Cecília de Oliveira Lessa, Domício Carvalho Lacerda, Carlos Eduardo Paiva, Marina Moreira Costa Zorzetto, Ana Julia Aguiar de Freitas, Iara Viana Vidigal Santana, Marco Antonio de Oliveira, Edenir Inêz Palmero, Márcia Maria Chiquitelli Marques, Tomás Reinert

**Affiliations:** 1 Barretos Cancer Hospital, Barretos, SP Brazil; 2 Molecular Oncology Research Center, Barretos Cancer Hospital, Teaching and Research Institute, Barretos, SP Brazil; 3 Pathology Department, Barretos Cancer Hospital, Barretos, SP Brazil; 4 Nucleus of Epidemiology and Biostatistics, Barretos Cancer Hospital, Barretos, SP Brazil; 5 Department of Genetics, Brazilian National Cancer Institute, Rio de Janeiro, Brazil; 6 Oncoclinicas, Porto Alegre, Brazil; 7 Grupo Brasileiro de Estudos em Câncer de Mama (GBECAM), Porto Alegre, Brazil

**Keywords:** Breast neoplasm, Neoadjuvant chemotherapy, Triple-negative breast cancer, BRCA

## Abstract

**Background:**

Neoadjuvant chemotherapy (NACT) is the mainstay of treatment of stages II and III triple-negative breast cancer (TNBC). This study aims to evaluate if the addition of carboplatin to NACT is associated with an increase in the pathological complete response (pCR) rates in TNBC.

**Methods:**

We conducted an open-label phase II randomized clinical trial in a single center in Brazil. Patients with stage II and III TNBC were randomized to receive standard NACT with or without carboplatin. All the patients received doxorubicin (60 mg/m^2^) plus cyclophosphamide (600 mg/m^2^) both intravenously (i.v.) q21 days for four cycles. Patients were then randomized for additional treatment with weekly (wk) paclitaxel (80 mg/m^2^ i.v., for 12 cycles) plus wk carboplatin AUC 1.5 (experimental arm) or without wk carboplatin (control arm). Randomization was stratified according to *gBRCA* status, age, and AJCC 8th edition clinical stage (II vs. III). The primary endpoint was the pathologic complete response (pCR) rate. Secondary endpoints included recurrence-free survival and overall survival.

**Results:**

Between 2017 and 2021, 146 patients were randomized, 73 on each arm. The median age was 45 years. Most patients (66.4%) had locally advanced stage III disease, 67.1% had T3/T4 tumors, and 56.2% had clinically positive axillary lymph nodes. Germline BRCA status was available for all patients, and 19.9% had pathogenic *BRCA*1/2 variants. The pCR rate (ypT0ypN0) was numerically increased by 13.7%, being 43.8% (31 of 73 patients) in the experimental and 30.1% (22 of 73 patients) in the control arm, not meeting the prespecified goal of increasing the pCR in 15% (*p*-value = 0.08). Survival outcomes are immature.

**Conclusion:**

The addition of carboplatin to standard NACT in stages II and III TNBC was associated with a non-statistically significant numerical increase in the pCR rate. Follow-up for survival outcomes and translational research initiatives are ongoing.

**Supplementary Information:**

The online version contains supplementary material available at 10.1007/s10549-023-07011-0.

## Introduction

Triple-negative breast cancer (TNBC) is a subtype that accounts for approximately 15–20% of all breast cancer diagnoses. Clinically defined as lacking ER, PR, and HER2 expression, TNBC is characterized by an aggressive natural history and worse survival outcomes compared with other breast cancer subtypes [1]. TNBC is more common in younger patients, African Americans, and BRCA-1 mutation carriers [2, 3].

Neoadjuvant chemotherapy (NACT) remains the mainstay of early-stage and locally advanced disease treatment, and pathological complete response (pCR) as a surrogate endpoint is well-established in TNBC [4]. Even though recent advances have allowed the incorporation of immunotherapy [5] and PARP-inhibitors [6] in the (neo)adjuvant treatment of TNBC, these advances are not available to most breast cancer patients in low- to middle-income countries (LMIC), where approximately 70% of global breast cancer deaths occur [7]. Therefore, optimizing the NACT is of great importance since clinical research findings evaluating routinely available chemotherapeutics can lead to advances with immediate incorporation into global clinical practice, even in the public health scenario.

Over the past years, there has been considerable interest in using platinum salts in treating TNBC because homologous recombination DNA repair dysfunction sensitizes tumor cells to these agents and induces cell death [3]. Recent studies have consistently demonstrated pCR gains by adding carboplatin to the standard NACT regimen based on anthracyclines and taxanes [4, 8]. Controversial results were seen in phase III studies, as some studies demonstrated consistent benefits in survival outcomes [9]. In contrast, other studies only showed pCR increase without disease-free survival (DFS) gain [10]. Notably, most of these data come from studies conducted in high-income countries. We have limited studies on molecular epidemiology and data from clinical trials conducted in LMIC patients, where a more significant proportion of locally advanced tumors and younger patients is present [2].

Therefore, we conducted a phase II randomized clinical trial to evaluate if adding carboplatin to standard NACT could increase the pCR rate in patients with known BRCA status presenting with early-stage and locally advanced TNBC in Brazil.

## Methods

NACATRINE is an open-label phase II randomized trial conducted in a single center in Brazil. The study was conducted in compliance with the guidelines of the Declaration of Helsinki, International Conference on Harmonization and Good Clinical Practice. All patients gave informed consent for using tissue and biomarker evaluation for research purposes. The NACATRINE trial was approved by the Medical Ethics Committee of Barretos Cancer Hospital (1.796.766) and was registered at ClinicalTrials.gov under the number NCT02978495. All patients signed voluntary informed consent before study entry.

### Patient population

Key eligibility criteria included patients more than 18 years old, with ECOG PS 0 or 1, adequate organ function with newly diagnosed stage II–III TNBC (ER < 1%, PR < 1%, and HER2 negative according to ASCO/CAP Guidelines [11] and no evidence of distant metastases. All patients had known germline BRCA1/2 mutational status. Bilateral TNBC should be confirmed by core biopsy in patients with bilateral tumors. Patients were excluded if they had a history of grade >/2 neuropathy, had previous treatment for breast cancer, and if pregnant or breastfeeding.

### Randomization and stratification

All eligible patients were randomized in a 1:1 ratio to receive standard NACT with or without carboplatin. Treatment was allocated by computerized local randomization using the REDCAP (Research Electronic Data Capture) software. Randomization was stratified according to *gBRCA* status (pathogenic/likely pathogenic variants vs. no pathogenic/ likely pathogenic variants), age (< 50 vs. ≥ 50 years), or AJCC 8th edition clinical stage (II vs. III).

### Procedures

All women were screened at baseline for distant metastasis with chest and abdomen tomography and bone scintigraphy. Blood samples were collected from all women at baseline to define BRCA1 and BRCA2 mutational status and storage at the BioBank of Barretos Cancer Hospital [12]. Genomic DNA was extracted from peripheral blood using the QIAmp DNA Blood Mini QIAcube Kit with the automated QIAcube (Qiagen) platform following the manufacturer’s instructions. *BRCA1/BRCA2* were sequenced using the NGS platforms Ion Torrent PGM or Illumina MiSeq System. Besides, the presence of rearrangements was evaluated through Multiplex Ligation-dependent Probe Amplification (MLPA). Pathogenic/likely pathogenic variants were confirmed in a new PCR reaction followed by conventional bi-directional sequencing (Sanger). Variant selection and classification were performed according to the criteria proposed by the American College of Medical Genetics and Genomics [13].

According to the investigator's choice, a sentinel node procedure was done in patients with clinically node-negative disease before or after NACT.

As summarized in Fig. S1 (supplementary material), the chemotherapy protocol consisted of doxorubicin (60 mg/m^2^) plus cyclophosphamide (600 mg/m^2^) both intravenously (i.v.) once every 21 days for four cycles for all patients. Patients were then randomized for additional treatment with paclitaxel (80 mg/m^2^ i.v.) once every 7 days for 12 cycles with carboplatin AUC 1.5 (experimental arm) once every 7 days for 12 cycles or without carboplatin (control arm).

An assessment of toxicity and laboratory tests preceded each chemotherapy cycle. Adverse events (AEs) were assessed according to Common Terminology Criteria for Adverse Events (CTCAE) version 4.03. Dose adjustment criteria followed the protocol. In brief, in the case of grade 2 neutropenia, chemotherapy was allowed with granulocyte-colony-stimulating factor (G-CSF) prophylaxis at the investigator’s discretion. In the case of grade 3 or 4 neutropenia, or febrile neutropenia, chemotherapy was delayed and postponed until grade 1 with dose reduction according to local protocol. For anemia, thrombocytopenia, and non-hematological toxicities grade 3 or worse, chemotherapy was postponed and reinitiated with dose reduction when toxicity recovered to grade 1. Toxicity-based dose adjustments were carried out according to drug-specific standard guidelines. In the experimental arm, if necessary, carboplatin was discontinued after two dose reductions, and paclitaxel was continued as monotherapy.

Patients underwent surgery within 3–6 weeks after the last chemotherapy cycle. The decision about performing breast-conserving surgery (BCS) or mastectomy depended on the patient’s and surgeon’s preferences and followed institutional guidelines. In patients with clinically node-positive disease after chemotherapy, axillary dissection was required. In patients with clinically node-negative disease, the timing (before or after neoadjuvant therapy) of sentinel node biopsy was at the investigator's local practice. Adjuvant radiotherapy was given according to local practice. All randomized patients remained in the study and follow-up.

### Outcomes

The primary endpoint was the pathologic complete response (pCR) rate. pCR was defined as no invasive tumor in the breast and lymph nodes (ypT0ypN0) and followed international guidelines [14]. Secondary endpoints were invasive DFS (iDFS), overall survival (OS), toxicity profile, and safety. iDFS was defined as the time from random assignment to invasive disease recurrence or death from any cause, and OS was defined as the interval from random assignment to death from any reason. Patients without an event were censored at the date of the last clinical assessment. All reported toxicities factored in the highest reported grade.

Additionally, biological samples were collected during the study to conduct molecular and clinical analyses to assess the presence of prognostic and predictive markers of benefit or resistance to the study regimens.

### Statistical analysis

We hypothesized that the carboplatin-containing neoadjuvant regimen could increase the pCR rate from 20 to 35% compared with the non-carboplatin neoadjuvant-containing regimen. Safety data were summarized descriptively for all patients who received at least one dose of study treatment. TEAEs leading to treatment interruption, dose reduction, or discontinuation of study drugs are reported.

For the sample description, the frequency was used for the qualitative variables and average and standard deviation for the quantitative ones. Comparison between groups was performed using the *χ*^2^, Fisher's Exact, and Mann–Whitney tests. Data normality was verified using the Kolmogorov–Smirnov tests. The odds ratio (OR) for the pCR rate was estimated by adjusting the Logistic Regression Model, and the estimated parameter's significance was verified using the Wald Test. An unadjusted log-rank test was used as the primary test to determine if there was a difference between the Kaplan–Meier survival curves. We used a Cox proportional hazards model as the primary treatment effect estimation alongside median survival with a 95% confidence interval (CI). The significance level adopted was 5%, and the analyses were performed using the IBM-SPSS v.27.0 software.

### Role of the funding source

The funder of the study had no role in study design, data collection, data analysis, data interpretation, or writing of the report.

## Results

Between 2017 and 2021, we screened for eligibility 154 patients and randomized 146 patients (73 in the carboplatin-containing regimen and 73 in the control group) in a single institution in Brazil. Figure S1 shows the consort study flow.

Patient characteristics were well-balanced between the two groups (as summarized in Table 1). The median age was 45 years, and 69.8% were younger than 50. Most patients (66.4%) had locally advanced stage III disease, 67.1% had T3/T4 tumors, and 56.2% had clinically positive axillary lymph nodes. 33.5% and 61.6% had histological grades II and III, respectively.

**Table 1:** Patients’ characteristics in the neoadjuvant chemotherapy arms of NACATRINE trial

Characteristic	Carboplatin-containing regimen (*n* = 73)	Non-carboplatin-containing regimen (*n* = 73)	*p* value
Age (years), *n* (%)			
< 50	50 (68.5)	52 (71.2)	0.857*
≥ 50	23 (31.5)	21 (28.8)	
Clinical stage (TNM) at baseline, *n* (%)			
II	24 (32.9)	25 (34.2)	0.99*
III	49 (67.1)	48 (65.8)	
Tumor size at baseline (cm), mean (SD)	7.18 (3.96)	6.19 (2.6)	0.31***
Tumor stage (T), *n* (%)			
cT1	0	1 (1.3)	0.933**
cT2	24 (32.9)	23 (31.5)	
cT3	29 (39.7)	31 (42.5)	
cT4	20 (27.4)	18 (24.7)	
Initial nodal status (N), *n* (%)			
cN0	34 (46.6)	30 (41.1)	0.617*
cN+	39 (53.4)	43 (58.9)	
Type of breast cancer surgery, *n* (%)			
Breast conserving surgery	20 (28.6)	26 (36.1)	0.373*
Mastectomy	50 (71.4)	46 (63.9)	
*BRCA* status, *n* (%)			
m *BRCA*	15 (20.5)	14 (19.2)	0.99*
wt *BRCA*	58 (79.5)	59 (80.8)	
Tumor grade, *n* (%)			
1	2 (2.8)	2 (2.8)	0.287**
2	20 (28.2)	29 (40.3)	
3	49 (69)	41 (56.9)	
Unknown			
Clinical response rate, *n* (%)			0.766*
Complete response	41 (56.2)	41 (56.2)	
Partial response	18 (24.7)	24 (32.8)	
Stable disease	4 (5.5)	4 (5.5)	
Progressive disease	6 (8.2)	4 (5.5)	

*OBS* there were one COVID-related death in the carboplatin arm and three patients with missing information about clinical response rate in the carboplatin arm (these patients where classified as non-pCR), *BRCA* breast cancer gene, *m BRCA* mutation *BRCA*, *wt BRCA* wild type *BRCA*, *TNM* classification of malignant tumors, *cm* centimeter, *SD* standard deviation, *N* number

*χ^2^ Test, **Exact Fisher test, ***Mann–Whitney test

Germline BRCA status was available for all patients, and 19.9% had pathogenic *BRCA1* or BRCA2 variants.

The breast-conservation surgery rate after NACT was 32.3%, and there were no differences in the mastectomy rate between the two groups: 71.4% in the carboplatin arm and 63.9% in the control arm (*p* = 0.373). Rates of objective clinical response were similar between the two groups (*p* = 0.76).

As described in Table 2, 54 (36.7%) patients enrolled in the study achieved pCR in the breast and the axillary lymph nodes. The pCR rate (ypT0ypN0) was numerically increased by 13.7%, being 43.8% (31 of 73 patients) in the carboplatin-containing regimen and 30.1% (22 of 73 patients) in the non-carboplatin-containing regimen, not meeting the prespecified goal of increasing the pCR in 15% (*p* value = 0.08).

**Table 2:** pCR rates according to treatment arm and *BRCA* germline mutation status

Patients characteristic	pCR		OR (95% CI)	*p* value
	Yes	No		
Arm, *n* (%)				
Paclitaxel	22 (30.1)	51 (69.9)	1	0.087
Paclitaxel + carboplatin	32 (43.8)	41 (56.2)	1.85 (0.91–3.75)	
*BRCA* status, *n* (%)				
WT	36 (30.8)	81 (69.2)	1	0.003
Mutation	18 (62.1)	11 (37.9)	3.74 (1.58–8.82)	

*WT* wild type, *pCR* pathological complete response, *OR* odds ratio, *CI* confidence interval, *N* number

The pCR rate was higher in patients with pathogenic BRCA mutation (62.1%) compared to patients with BRCA wild-type status (30.8%): OR 3.74 (95% CI 1.58–8.82, *p* = 0.003, Table 3). The interaction test between carboplatin and *BRCA* mutation revealed nonsignificant results (OR 2.75, 95% CI 0.58–12.98, *p* = 0.201).

**Table 3:** Comparison of pCR by treatment arms and by *BRCA* status

Type of treatment in carboplatin vs. non-carboplatin	pCR	
	OR (95% CI)	*p* value
Carboplatin vs. non-carboplatin, *BRCA* mutation	2.75 (0.58–12.98)	0.201
Carboplatin vs. non-carboplatin, *BRCA* wild type	1.67 (0.75–3.68)	0.208

*pCR* pathological complete response, *OR* odds ratio, *CI* confidence interval

**Fig. 1:**
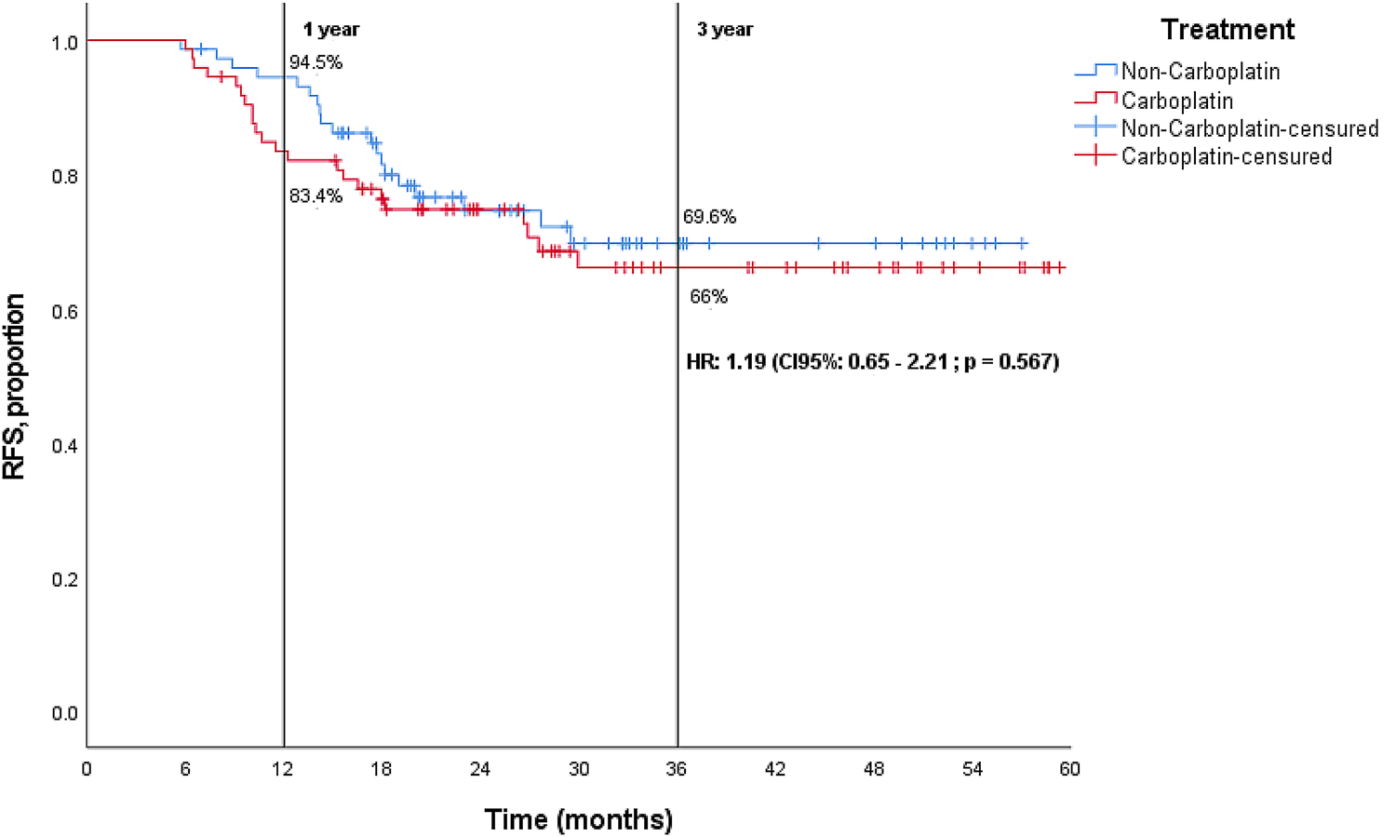
Disease-free survival in intention-to-treat population

**Fig. 2:**
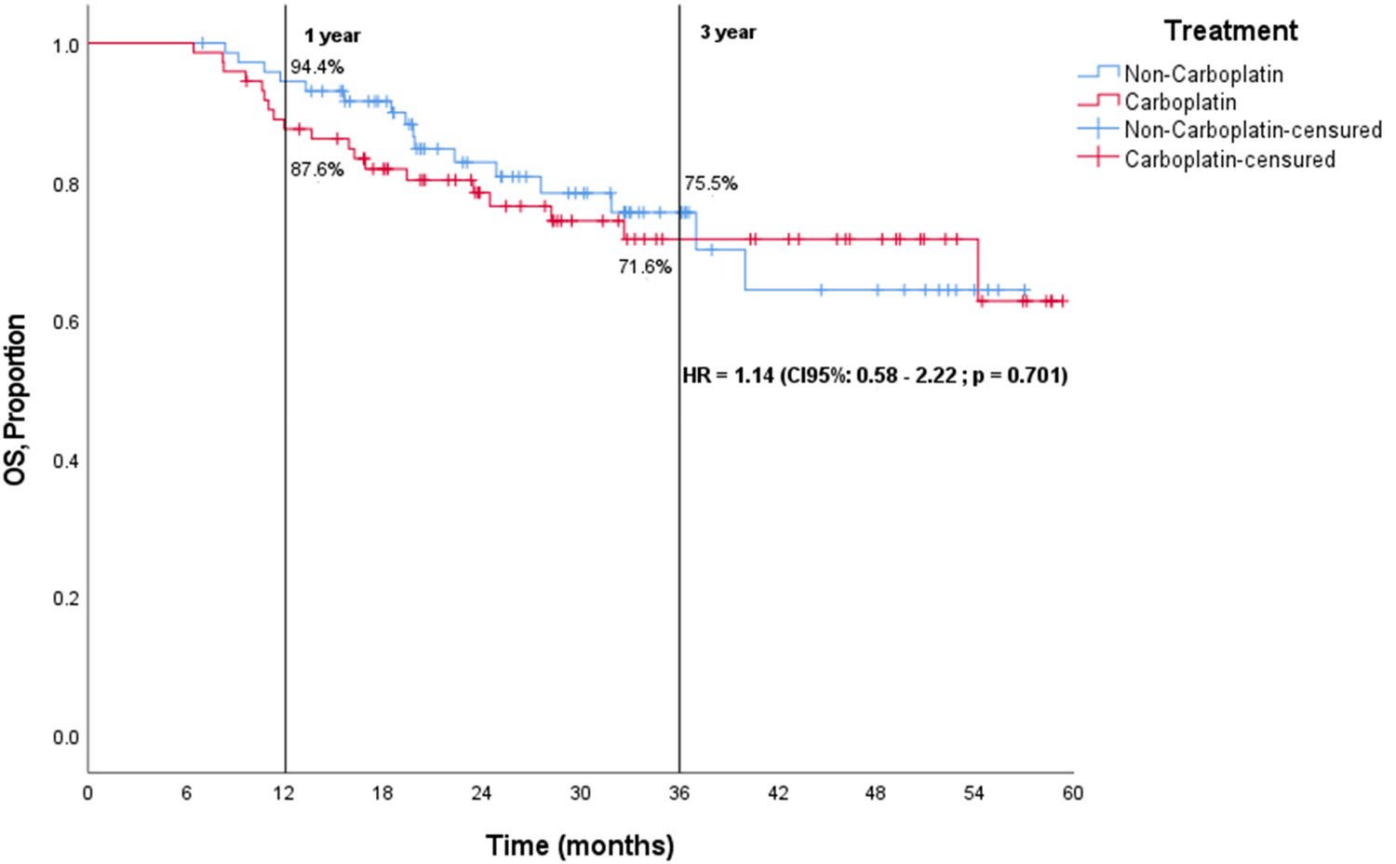
Overall survival (OS) in intention-to-treat population

The median RFS (Fig. 1) and median OS (Fig. 2) were not reached, with a median follow-up of 47.7 months. At 3 years, there was no difference in the survival outcomes between the two treatment arms. The proportion of patients without recurrence was 66% in the carboplatin-containing regimen, and 69.6% in the non-carboplatin-containing regimen [HR = 1.19 (95% CI 0.65–2.21, *p* = 0.567)], and the proportion of patients alive was 71.6% in the carboplatin-containing regimen and 75.5% in the non-carboplatin-containing regimen [HR = 1.14 (95% CI 0.58–2.22, *p* = 0.701)].

Most of the relevant toxicities reported during NACT in this clinical trial are summarized in Table 4. The safety and toxicity profiles of the patients included in this trial were similar and comparable to other randomized clinical trials of neoadjuvant carboplatin and mirrored what is usually seen in routine clinical practice. All patients received the initial four cycles of AC, and there were no significant toxicity issues in this part, and the frequency of adverse effects was similar between the two arms. In the taxane (with or without carboplatin) part of the protocol, the most common AEs in both groups were nausea, fatigue, neuropathy, anemia, and neutropenia. Hematological toxicity, primarily neutropenia, was higher in the carboplatin arm. However, the incidence of febrile neutropenia was low (one patient in each arm). Non-hematological toxicities such as nausea, fatigue, neuropathy, and mucositis were similar between the two arms. Notably, 4 patients (5.5%) in the carboplatin arm experienced grade 3 peripheral neuropathy.

**Table 4:** Haematological and non-haematological adverse effects

	Paclitaxel + carboplatin		Paclitaxel	
Adverse event	G1–2	G ≥ 3	G1–2	G ≥ 3
Anemia, *n* (%)	63 (86.3)	1 (1.4)	55 (75.3)	1 (1.4)
Neutropenia, *n* (%)	47 (64.4)	11 (15.1)	27 (37)	8 (11)
Thrombocytopenia, *n* (%)	5 (6.8)	0	2 (2.7)	0
Febrile neutropenia, *n* (%)	4 (5.5)	1 (1.4)	1 (1.4)	0
Nausea, *n* (%)	56 (76.7)	0	57 (78.1)	0
Vomiting, *n* (%)	18 (24.7)	0	22 (30.1)	0
Fatigue, *n* (%)	58 (79.5)	1 (1.4)	56 (76.7)	0
Mucositis, *n* (%)	20 (27.4)	0	21 (28.8)	0
ALT/AST increased, *n* (%)	16 (21.9)	0	14 (19.2)	0
Neuropathy, *n* (%)	31 (42.5)	4 (5.5)	31 (42.5)	0
Pruritus, *n* (%)	16 (21.9)	1 (1.4)	22 (30.1)	0
Rash, *n* (%)	(19.2)	0	20 (27.4)	0

As summarized in Table 5, dose reductions were more frequent in the carboplatin arm (30.1% vs. 9.6%, *p* = 0.003), most often due to hematological toxicity. However, there was no difference in the rate of permanent treatment discontinuation between groups (34.2% vs. 32.1%, *p* = 0.488).

**Table 5:** Frequency of dose reductions, reasons for dose reduction and permanent treatment discontinuations

	Experimental arm	Control arm	*p* value
	Paclitaxel + carboplatin (*n* = 73)	Paclitaxel (*n* = 73)	
Treatment dose reduction, *n* (%)			
No	51 (69.9)	66 (90.4)	
Yes	22 (30.1)	7 (9.6)	0.003*
Reasons for dose reduction, *n* (%)			
Hematology toxicity	19 (26)	7 (9.5)	
Non-hematology toxicity	3 (4.1)	0	
Treatment discontinuation, *n* (%)	13 (34.2)	9 (32.1)	0.488*

*χ^2^ Test

## Discussion

In the NACATRINE study, a phase II randomized clinical trial conducted at a single center in Brazil, the addition of carboplatin to an anthracycline and taxane-based NACT regimen was associated with a numerical but not statistically significant increase in the pCR rate. Survival data are immature and, so far, do not show differences between groups. The toxicity profile was favorable and comparable to previous data, with increased neutropenia rate and dose reductions. However, the incidence of severe adverse effects was low, and there was no difference in treatment interruption between the two groups.

NACT remains the standard treatment for early-stage and locally advanced TNBC. Platinum cytotoxic agents (such as carboplatin) cause DNA strand breaks via cross-linkage of DNA strands, increasing their effectiveness in tumors with impaired DNA repair pathways, a finding commonly seen in TNBC as well as in patients with hereditary mutations such as BRCA or other homologous recombination pathways genes [15]. Hence, various clinical and translational research initiatives have focused on the role of platinum-based chemotherapy for TNBC. When the NACATRINE study was designed, this was a controversial topic as studies consistently demonstrated pCR gains, but the benefit on survival outcomes was not shown in most trials. Subsequently, recent studies, such as the phase III BRIGHTNESS clinical trial and meta-analyses involving individual patient data, have demonstrated significant DFS and OS gains and practically confirmed the role of carboplatin in the neoadjuvant treatment of TNBC [9]. Consequently, regimens containing combinations of anthracyclines, taxanes, and carboplatin are standard of care in routine clinical practice and used as the chemotherapy backbone in contemporary trials evaluating innovative agents.

Benefits in terms of pCR and survival outcomes have placed the Keynote-522 study protocol, containing polychemotherapy with carboplatin associated with the immunotherapy pembrolizumab, as the preferred regimen for the neoadjuvant treatment of stages II and III TNBC [5]. However, immunotherapy is not accessible to most breast cancer patients in developing countries. Consequently, the optimization of the chemotherapy protocol remains an important issue. Although recent studies show a tendency towards the incorporation of carboplatin into NACT, it is essential to emphasize that most of these studies were conducted in developed countries, and we do not have adequate data on this subject in women from developing countries with TNBC, a population with potentially distinct epidemiological characteristics and that more often present at a younger age, with aggressive and locally advanced tumors. This study was conducted in a single institution, Barretos Cancer Hospital (Barretos, SP, Brazil), an oncology referral center that exclusively serves patients from the public health system. Therefore, patient profiles, as well as care and treatment routines, adequately reflect the context of LMIC's oncology services.

Genetic diversity of germline variants in TNBC predisposition genes is unexplored in miscegenated populations, such as those living in Latin America [16]. A positive fact in our study is that all patients had known BRCA status, something challenging to be accomplished in the context of LMIC. In Brazil, we need more data on the molecular epidemiology of TNBC. The information that 20% of Brazilian women with TNBC stages II and III carry germline pathogenic variants in BRCA1 or BRCA2 is important from an academic perspective and the point of view of health policy planning and institutional guidelines, as these women may be candidates for new targeted therapies, such as the use of the PARP inhibitor olaparib in patients with post-NACT residual disease [6].

Our findings are comparable to the published literature in the field, considering that various randomized clinical trials consistently demonstrated increased clinical response and pCR rates using neoadjuvant carboplatin in TNBC. The relationship between the use of neoadjuvant carboplatin and long-term survival benefits has been considered a controversial issue in breast oncology, considering that many studies that demonstrated pCR gain did not show significant benefits in terms of DFS and OS. However, recently the BRIGHTNESS study was published [8, 9], revealing a DFS gain (HR 0.63, *p* = 0.02) and a significant increase of 19% in pCR (58% carboplatin arm vs. 31% non-carboplatin arm), which was slightly higher than the rate we found in our study (13.7%; 43.8% carboplatin arm vs. 30.1% non-carboplatin arm; *p* = 0.08). Importantly, in our study, patients with a pathogenic BRCA mutation presented a significantly higher pCR rate compared to patients with BRCA wild-type status (62.1% vs. 30.8%, respectively); however, no interaction between the presence of BRCA mutation and carboplatin use was identified. A recently published meta-analysis with individual patient data from eight trials enrolling 2425 patients reported that carboplatin improved DFS (HR 0.66; 95% CI 0.55 to 0.80, *p* < 0.001) and OS (HR 0.68; 95% CI 0.54 to 0.87, *p* = 0.002). The pCR rate was better in the carboplatin arm (OR 2.11; 95% CI = 1.44–3.08; I2 67%, *p* = 0.009), as expected [17]. In a phase III randomized clinical trial from India study presented in SABCS 2023, the use of neoadjuvant carboplatin increased pCR rates (54.5% versus 40.3%, *p* < 0.01) and was associated with a statistically significant DFS benefit (5-year DFS 74.2% platinum group and 61.7% in the control arm, HR 0.64–*p* = 0.004) in younger patients [18]. Therefore, the incorporation of carboplatin into the standard NACT regimen is now considered standard of care and recommended by international guidelines [19].

The present study adds to the growing body of evidence supporting carboplatin as a component of NACT in stage II–III TNBC. We acknowledge that our study has several limitations, including the absence of dose-dense chemotherapy given the lack of access to colony-stimulating factors (G-CSF) in the public health system of LMIC, the relatively small sample size and difficulties inherent to the lack of statistical power in a phase II study. Despite not crossing the threshold for statistical significance of 15%, we believe the increase in the pCR rate observed in this study can be considered clinically significant and congruent with other randomized clinical trials. Since carboplatin is a widely accessible treatment even in LMIC, with low cost and a known and manageable toxicity profile, we consider that it can be regarded as in most cases of patients with TNBC with an indication for NACT in current clinical practice.

Our study has several qualities and opportunities, such as an adequate clinical-epidemiological description of the profile of patients with TNBC treated in a public health institution in a LMIC, in addition to providing results in terms of pathological response and survival outcomes that have diverse applicability, including for planning new clinical research initiatives. All patients included had samples collected for a well-structured biobank that will serve for translational research initiatives, given that identifying predictive biomarkers to better define subsets of TNBC patients who benefit from the addition of carboplatin remains an unmet need. Additionally, the study aimed to identify biomarkers associated with pCR, residual invasive disease after NACT, and recurrence, and we intend to publish these exploratory analyses in the future.

## Conclusion

The addition of carboplatin to standard NACT in stages II and III TNBC was associated with a non-statistically significant numerical increase in the pCR rate of 13.7%, consistent with other similar clinical trials. Follow-up for survival outcomes and translational research initiatives are ongoing. Given the consistent results with previous studies, the addition of carboplatin appears to have a favorable risk-to-benefit profile. It might be considered a potential NACT component for patients with high-risk TNBC in LMIC.

## Supplementary Information

Below is the link to the electronic supplementary material.

Supplementary file1 (DOCX 47 KB)

## Data Availability

The data generated in this study are available upon request from the corresponding author.
