# Supplementary material for: Neoadjuvant carboplatin in triple-negative breast cancer: results from NACATRINE, a randomized phase II clinical trial
Source: Breast Cancer Res Treat. 2023 Aug 14;202(1):57–65. doi: 10.1007/s10549-023-07011-0 (PMC10504209; doi:10.1007/s10549-023-07011-0)
Supplement: Supplementary file 1 — Supplementary file1 (DOCX 47 KB) [file 10549_2023_7011_MOESM1_ESM.docx]

**Figure S1.** Consort study flow.
